# Presumed stromal graft rejection after automated lamellar therapeutic keratoplasty: case report

**DOI:** 10.1186/1752-1947-1-10

**Published:** 2007-04-01

**Authors:** Motoko Kawashima, Hiroshi Mochizuki, Tetsuya Kawakita, Shin Hatoh, Jun Shimazaki, Masakazu Yamada

**Affiliations:** 1 Department of Ophthalmology, Tokyo Dental College, Chiba, Japan; 2 Division for Vision Research, National Institute of Sensory Organs, National Tokyo Medical Center, Tokyo, Japan

## Abstract

**Purpose:**

To describe the development of presumed immune-mediated stromal rejection after automated lamellar therapeutic keratoplasty (ALTK) and its reversal after initiation of intensive topical corticosteroid therapy.

**Methods:**

Observational case report.

**Results:**

Stromal edema localized in the graft developed 42 days after ALTK for Avellino corneal dystrophy in a 65-year-old man. After one week of intensive topical corticosteroids, complete reversal of graft edema occurred, with full recovery of visual function.

**Conclusion:**

The clinical appearance and response to therapy in this case supported the diagnosis of immune-mediated stromal rejection. Ophthalmologists should be aware that stromal rejection may occur in lamellar corneal grafts.

## Background

Lamellar keratoplasty was the first form of corneal transplantation ever attempted, and now has a history of over a century. Occasionally, it is employed in the rehabilitation of thinned corneas or those with anterior opacification[1]. However, the use of lamellar keratoplasty has been limited by difficulties such as irregularity and scarring of tissue interfaces, leading to poor visual outcomes compared with penetrating keratoplasty, as well as technical difficulties and prolonged operating time[1]. The thickness and contour of the transplanted tissue are difficult to control, which causes problems with optical clarity.

Automated lamellar therapeutic keratoplasty (ALTK) is a new approach to lamellar keratoplasty which avoids some of these problems[2]. In ALTK, an automated keratome is used to cut partial-thickness sections through the anterior surfaces of both the donor and host corneas. These sections are very similar to the flaps cut in LASIK surgery, and allow a very precise surface to be obtained. Thus, ALTK offers advantages over traditional lamellar keratoplasty, as it reduces astigmatism, thereby resulting in better potential visual outcomes and shorter operation time.

Lamellar grafting offers several advantages over penetrating keratoplasty, including elimination of allograft rejection and avoidance of intraocular complications. Although there is still the possibility of rejection at either the donor epithelium or stroma, the avoidance of endothelial rejection is an exceptional advantage. Irreversible loss of vision after lamellar keratoplasty due to presumed stromal rejection has occurred in only 1.4%–1.9% of patients according to previous reports[3,4]. This lower rate of stromal rejection may due to the small number of lamellar keratoplasties performed compared to penetrating keratoplasties, and an increased number of lamellar keratoplasties may, therefore, result in an increased number of stromal rejection. Here, we report a case of presumed immune-mediated stromal rejection after ALTK that was completely reversed with prompt initiation of intensive steroid therapy.

## Case presentation

A 65-year-old Japanese man was referred to the National Tokyo Medical Center because of blurred vision in both eyes. The patient was diagnosed with Avellino corneal dystrophy, and had a history of keratectomy in both eyes at the age of 60. Corrected visual acuity was 20/100 in the right eye, and 20/50 in the left eye. Examination by slit-lamp microscopy revealed gray-white granular opacities in the anterior stroma of both eyes, which was compatible with the diagnosis of Avellino corneal dystrophy. The patient's medical history was otherwise unremarkable. His daughter also had Avellino corneal dystrophy.

Additional PTK was ruled out because of insufficient corneal thickness due to previous keratectomy, and ALTK was performed in the right eye to remove anterior stromal deposit in October, 2004. Using the "Moria LSK Evo-II MircoKeratome Evo II Micro Keratome (Moria Japan, Tokyo, Japan), a 9.5-mm-diameter, 200 μm-depth flap was cut out from the recipient cornea. In same way, a 9.5-mm-diameter, 300 μm flap was obtained from a donor cornea maintained in an artificial chamber (Moria Japan). The donor cornea was transported from an eye bank in the United States, and met the criteria of the Eye Bank Association of America for donor quality. Fresh, full-thickness graft material preserved in Optisol GS (Bausch & Lomb, Rochester, NY) for 7 days was used for the lamellar keratoplasty. The lamellar graft was sutured in place with 9 interrupted 10-0 nylon sutures. Topical betamethasone phosphate 0.1% (Shionogi Pharmateutical Co., Osaka, Japan) and levofloxacin 0.5% (Santen Pharmateutical Co., Osaka, Japan) were applied three times daily in the postoperative periods. Visual acuity improved to 20/40 with correction at 4 weeks after the operation.

**Figure 1:**
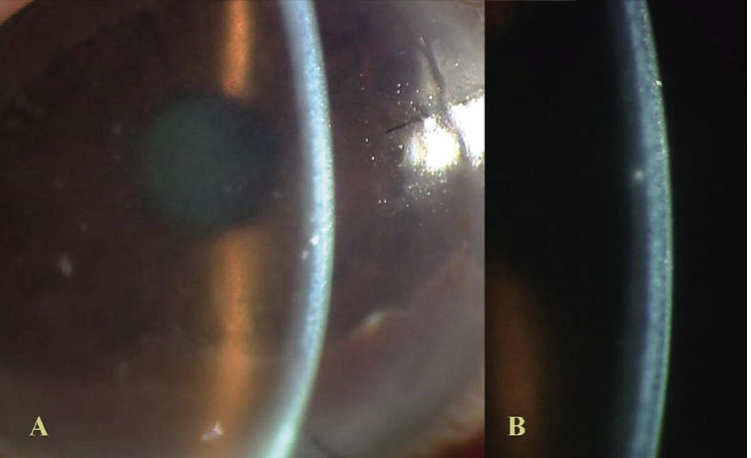
Day 42 after surgery. A. Diffuse edema of lamellar graft was apparent, whereas there were no epithelial defects, keratic precipitates, or inflammation in anterior chamber. Gray-white granular opacities were observed in the graft bed, which were residual deposits of Avellino corneal dystrophy. B. High-magnification slit-lamp photograph demonstrated marked anterior stromal thickening (graft edema). In contrast, posterior half of corneal stroma, which was recipient bed, remained clear.

**Figure 2:**
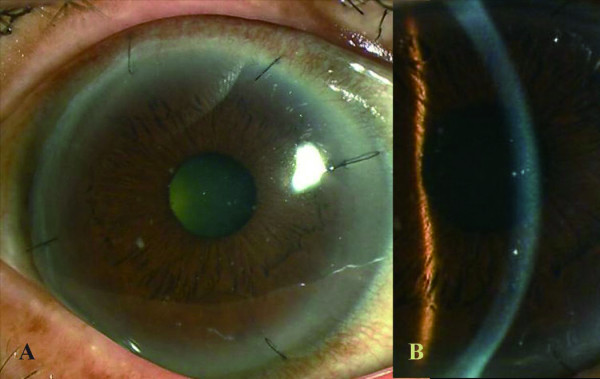
One week after treatment with topical corticosteroids. Corneal stroma cleared completely.

Forty-two days postoperatively, the patient returned to our clinic with the sensation of a foreign body and blurred vision in the right eye. Slit-lamp examination revealed a diffuse stromal edema limited to the graft (Fig. 1). The posterior half of the corneal stroma, which was the recipient bed, remained clear. There were no epithelial defects, and no keratic precipitates or inflammation were seen in the anterior chamber. Intraocular pressure was 12 mmHg. A diagnosis of presumed immune-mediated stromal rejection was made based on these findings. On an hourly regimen of betamethasone phosphate 0.1%, the stromal edema began to improve immediately, completely clearing within 1 week (Fig. 2). Corrected visual acuity in the right eye improved to 20/25 at 10 days after treatment. Topical corticosteroids were tapered and discontinued over 3 months, and the graft remained clear at the last follow-up.

## Discussion

The patient developed stromal edema localized in the graft 42 days after ALTK. Differential diagnoses might have included diffuse lamellar keratitis (DLK) and herpetic keratitis, as well as allograft rejection. DLK is usually a postoperative complication of LASIK in early phase (within 1–6 days after surgery), and is defined as an inflammatory condition in which white blood cells migrate along the stromal interface[5]. The etiology of DLK remains to be clarified, and it is generally thought that the cause of this inflammatory reaction may be multifactorial [6].

In this case, DLK could be excluded due to the lateness of onset (42 days after the operation) and involvement of the entire graft, not just the interface. Herpetic infection could also be excluded due to the localization of the stromal edema in the graft and negative history of herpetic eye infection. Although stromal edema has been reported to sometimes occur following LASIK, such as in eyes with uveitis and elevated intraocular pressure[7], this possibility was also excluded due to the maintenance of normal range (around 12 mmHg) throughout the follow-up period. The absence of associated ocular abnormalities and the prompt response to intensive corticosteroid therapy indicated a diagnosis of stromal allograft rejection after ALTK. Stromal rejection involves infiltration rather than edema of the stroma[8], and recently Watson et al reported that the stroma became opaque and edematous in 2 stromal rejection cases after deep lamellar keratoplasty[4], which also supported our diagnosis. In our case, one contributing factor to stromal rejection could be the large graft size in ALTK

Three different types of allograft rejection have been identified after penetrating keratoplasty: endothelial rejection, epithelial rejection, and subepithelial infiltrates [9]. Theoretically, lamellar grafts are free from endothelial rejection, but not from other types of rejection. Alldredge and Krachmer[9] reported that frequencies of epithelial rejection and subepithelial infiltrates after penetrating keratoplasty were 10% and 15%, respectively. However, the true frequencies of these two types of rejection are hard to determine because they can easily take place without symptoms between examinations. In our case, the patient developed a diffuse stromal edema localized in the graft, with no epithelial involvement, which ruled out epithelial rejection or subepithelial infiltrates.

Our case appears to resemble the case of stromal rejection after deep lamellar keratoplasty described by Al-Torbak and associates[10]. In their case, diffuse stromal edema of the entire graft developed 16 months after surgery for keratoconus. Although such stromal rejection is not included in reported criteria of allograft rejection after penetrating keratoplasty[9], it might be overshadowed by endothelial rejection in penetrating keratoplasty.

## Conclusion

In this case, the clinical appearance and response to therapy supported the diagnosis of immune-mediated stromal rejection. This case suggests that stromal rejection can occur after lamellar keratoplasty and that it usually goes unrecognized. We propose that ophthalmologists should be aware of stromal rejection as a potential complication of lamellar corneal grafts.

## Abbreviations

ALTK; automated lamellar therapeutic keratoplasty, PTK; phototherapeutic keratectomy, DLK; diffuse lamellar keratitis, LASIK; Laser in situ keratomileusis.

## Authors' contributions

MK drafted the manuscript. TK and JS helped to draft the manuscript. SH and HM participated in the data collection and treat this case. MY performed surgery and treat this case. All authors read and approved the final manuscript.
